# Clinical phenotypes and prognostic factors in persons with hip osteoarthritis undergoing total hip arthroplasty: protocol for a longitudinal prospective cohort study (HIPPROCLIPS)

**DOI:** 10.1186/s12891-023-06326-9

**Published:** 2023-03-25

**Authors:** Abner Sergooris, Jonas Verbrugghe, Thomas Matheve, Maaike Van Den Houte, Bruno Bonnechère, Kristoff Corten, Katleen Bogaerts, Annick Timmermans

**Affiliations:** 1 REVAL Rehabilitation Research, Faculty of Rehabilitation Sciences, Hasselt University, Agoralaan Building A - B-3590, Diepenbeek, Belgium; 2 Spine, Head and Pain Research Unit Ghent, Department of Rehabilitation Sciences, Ghent University, Ghent, Belgium; 3 Laboratory for Brain-Gut Axis Studies (LABGAS), Translational Research Center for Gastrointestinal Disorders (TARGID), Department of Chronic Diseases and Metabolism, University of Leuven, Leuven, Belgium; 4 Department of Orthopaedics – Hip Unit, Ziekenhuis Oost-Limburg, Genk, Belgium; 5 Centre for Translational Psychological Research (TRACE), Ziekenhuis Oost-Limburg, Genk, Belgium; 6 Health Psychology, Faculty of Psychology and Educational Sciences, KU Leuven, Leuven, Belgium

**Keywords:** Clinical phenotype, Biopsychosocial model, Hip osteoarthritis, Total hip arthroplasty, Prognostic factors, Trauma, Mental disorders, Quantitative sensory testing, Pain

## Abstract

**Background:**

Large heterogeneity exists in the clinical manifestation of hip osteoarthritis (OA). It is therefore not surprising that pain and disability in individuals with hip OA and after total hip arthroplasty (THA) cannot be explained by biomedical variables alone. Indeed, also maladaptive pain-related cognitions and emotions can contribute to pain and disability, and can lead to poor treatment outcomes. Traumatic experiences, mental disorders, self-efficacy and social support can influence stress appraisal and strategies to cope with pain, but their influence on pain and disability has not yet been established in individuals with hip OA undergoing THA. This study aims (1) to determine the influence of traumatic experiences and mental disorders on pain processing before and shortly after THA (2) to identify preoperative clinical phenotypes in individuals with hip OA eligible for THA, (3) to identify pre- and early postoperative prognostic factors for outcomes in pain and disability after THA, and (4) to identify postoperative clinical phenotypes in individuals after THA.

**Methods:**

This prospective longitudinal cohort study will investigate 200 individuals undergoing THA for hip OA. Phenotyping variables and candidate prognostic factors include pain-related fear-avoidance behaviour, perceived injustice, mental disorders, traumatic experiences, self-efficacy, and social support. Peripheral and central pain mechanisms will be assessed with thermal quantitative sensory testing. The primary outcome measure is the hip disability and osteoarthritis outcome score. Other outcome measures include performance-based measures, hip muscle strength, the patient-specific functional scale, pain intensity, global perceived effect, and outcome satisfaction. All these measurements will be performed before surgery, as well as 6 weeks, 3 months, and 12 months after surgery. Pain-related cognitions and emotions will additionally be assessed in the early postoperative phase, on the first, third, fifth, and seventh day after THA. Main statistical methods that will be used to answer the respective research questions include: LASSO regression, decision tree learning, gradient boosting algorithms, and recurrent neural networks.

**Discussion:**

The identification of clinical phenotypes and prognostic factors for outcomes in pain and disability will be a first step towards pre- and postoperative precision medicine for individuals with hip OA undergoing THA.

**Trial registration:**

ClinicalTrials.gov: NCT05265858. Registered on 04/03/2022.

## Background

Hip osteoarthritis (OA) is one of the leading causes of pain and disability [1–4]. Each year, more than 40 million prevalent cases and more than 2 million incident cases are reported worldwide [3]. The global prevalence and incidence are expected to increase considerably in the upcoming decades, as a result of the aging population and an increasing prevalence of risk factors such as obesity and sedentary lifestyle [1, 5]. Large heterogeneity exists in the clinical manifestation of hip OA, which can be explained by its multifactorial nature and the comorbidities involved [1]. It is therefore not surprising that current conservative treatments according to a one-size-fits-all approach have small to moderate effectiveness [6–8]. Corresponding to the increasing prevalence and incidence of hip OA, the number of total hip arthroplasties (THA) is growing considerably [9]. The mean occurrence rate of hip implants in countries belonging to the Organisation for Economic Co-operation and Development is expected to increase from 145 in 2010 to 275 per 100.000 inhabitants in 2050 [9]. Although THA is considered highly successful in terms of prosthesis-related outcomes, previous studies reported that up to 23% of individuals report long-term pain following THA [10], and over 30% of individuals report limitations in activities of daily living two years after THA [11].

The identification of clinical phenotypes and prognostic factors in individuals with hip OA undergoing THA may help to unravel the complex and heterogeneous nature of hip OA and the prognosis after THA. Clinical phenotypes can be described as subgroups within a given population that are characterized and identified based on a collection of shared clinical characteristics [12, 13]. Several phenotype classifications based on clinical characteristics such as pain sensitization, psychological comorbidities, and radiographic severity have already been found in individuals with knee OA [12, 14]. However, the evidence for clinical phenotypes in individuals with hip OA is limited and has mainly focussed on variables within one domain of the biopsychosocial model [15, 16]. Furthermore, no studies have investigated the existence of clinical phenotypes in individuals after THA for hip OA. The identification of clinical phenotypes in individuals with hip OA undergoing THA can be a first step towards pre- and postoperative precision medicine, as it will allow the development of targeted treatments for specific subgroups [14, 17]. Additionally, prognostic factors for outcomes in pain and disability can inform treatment decisions in individuals eligible for THA, which can be an important step towards the improvement of outcomes after THA [18]. In the current body of research, studies have mainly focused on preoperative candidate prognostic factors and have not considered the role of early postoperative factors in the prognosis of outcome after THA. Biomedical variables, such as comorbidities and radiographic severity, have already been identified as prognostic factors for pain and disability after THA [19, 20]. However, pain and disability in individuals with hip OA and after THA cannot be explained by biomedical variables alone [21].

Indeed, maladaptive pain-related cognitions, emotions, and behavioural factors are known contributors to pain and disability in individuals with musculoskeletal pain, and can lead to poor treatment outcomes [22–30]. Preoperative fear-avoidance behaviour is an important prognostic factor for outcomes after knee and hip surgery [31, 32]. Furthermore, increasing evidence suggests that cognitive appraisals such as perceived injustice contribute to pain and disability in individuals with musculoskeletal disorders, including non-traumatic conditions such as OA [33–36]. Additionally, other psychological and social factors can influence the threat appraisal of pain and the ability to cope with stressful life situations. Evidence suggests that self-efficacy and social support may be associated with the development of disability and the ability to cope with pain [37–39]. Childhood trauma is known to be associated with a higher risk of chronic pain in adulthood, including OA-related pain [40, 41]. Furthermore, traumatic experiences can lead to a significant increase in the risk for mood and anxiety disorders [42, 43]. Symptoms of anxiety are highly prevalent among individuals with hip OA [44] and are known to be associated with higher levels of pain and disability in persons with hip OA and after THA [45]. However, the role of traumatic experiences and mental disorders in the pain processing of individuals with hip OA undergoing THA remains unknown. Furthermore, despite their importance in musculoskeletal disorders, no studies have investigated the existence of clinical phenotypes based on the aforementioned biopsychosocial characteristics, and the prognostic role of factors such as perceived injustice and traumatic experiences has not yet been investigated in individuals undergoing THA for hip OA.

Finally, the heterogeneity in the clinical presentation of individuals with hip OA and in the outcomes after THA may be explained by the pain mechanisms involved [46]. Increasing evidence suggests that central mechanisms play an important role in the pain experience of individuals with hip OA [47]. Widespread hyperalgesia, increased temporal summation of pain, and altered pain modulation through descending inhibitory pain pathways are known indicators of this central involvement [46]. Mechanistic pain profiling using quantitative sensory testing (QST) has already been used to profile individuals with OA based on involved pain mechanisms [48] and is argued to be a promising method to predict the outcomes of pain and disability after total joint arthroplasty [49, 50]. However, the current body of research has not considered both self-reported biopsychosocial variables and mechanistic pain profiling to identify clinical phenotypes in individuals with hip OA undergoing THA.

Therefore, in line with the above identified knowledge gaps, the following objectives were defined: (1) to determine the influence of traumatic experiences and mental disorders on the pain processing of individuals with hip OA and shortly after THA, (2) to identify pre-operative clinical phenotypes in individuals with hip OA and compare their prognosis for outcomes in pain and disability after THA, (3) to identify pre- and early postoperative prognostic factors for long-term outcomes in pain and disability after THA for hip OA, and (4) to identify postoperative clinical phenotypes based on biopsychosocial characteristics at different timepoints up to one year after THA for hip OA, and their pre- and early postoperative prognostic factors.

## Methods

### Study design

**Fig. 1:**
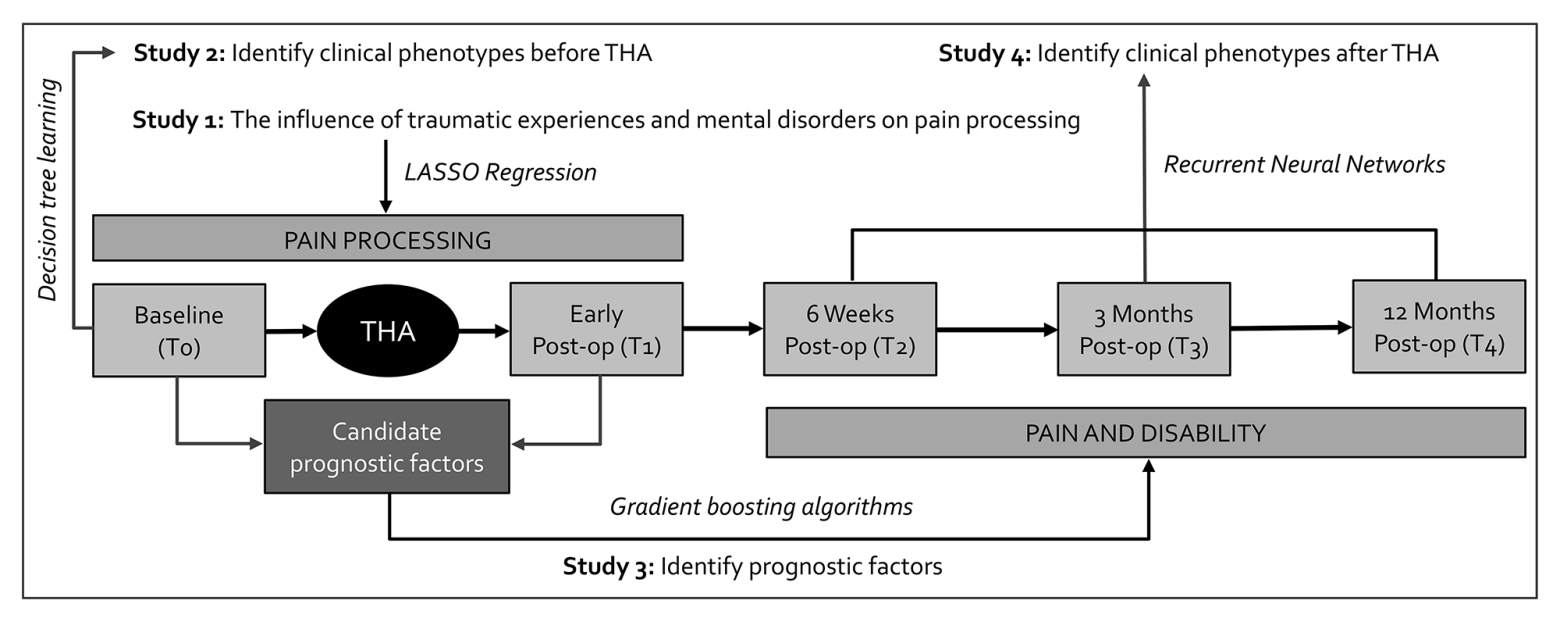
Timeline and schematic overview of the HIPPROCLIPS-trial. Legend: Main statistical methods are in italic. THA = total hip arthroplasty

A longitudinal prospective cohort study will be conducted to identify clinical phenotypes and prognostic factors for outcomes in pain and disability in individuals with hip OA undergoing THA (HIPPROCLIPS, ClinicalTrials.gov Identifier: NCT05265858). Ethical approval was obtained from the medical ethical committee of Hospital East-Limburg and Hasselt University (B3712021000002). Measurements will be performed at five major timepoints: one week before THA (T0), during the first week after THA (T1), and six weeks (T2), three months (T3), 12 months after THA (T4). During the first week after THA, participants will be asked to complete a minor set of questionnaires on the first, the third, the fifth, and the seventh postoperative day. A timeline and schematic overview of the HIPPROCLIPS-trial can be found in Fig. 1. The HIPPROCLIPS-trial is divided in four sub-studies based on cross-sectional and longitudinal analyses, details on the different studies can be found in Table 1.

**Table 1:** Overview of the HIPPROCLIPS-trial studies

Study	Objective	Research Questions (RQ)	Phenotyping variables or candidate prognostic factors	Outcome variables	Design and main statistical method
1	To determine the influence of traumatic experiences and mental disorders on the pain processing of individuals with hip OA and shortly after THA	1. To which extent are traumatic experiences and mental disorders associated with preoperative pain processing in individuals with hip OA waiting for THA? 2. To which extent are traumatic experiences and mental disorders associated with pre- and early postoperative pain-related cognitions and emotions in individuals with hip OA undergoing THA?	- Traumatic experiences (T0) - Mental disorders (T0)	Primary: QST (T0) Secondary: Pain-related cognitions and emotions, other pain-related variables (T0, T1)	**Design**: cross-sectional (RQ1) and longitudinal analysis (RQ2) **Main statistical method**: LASSO regression
2	To identify preoperative clinical phenotypes in individuals with hip OA and their prognosis for outcomes in pain and disability after THA	1. Which clinical phenotypes exist in individuals with hip OA waiting for THA, based on a set of biopsychosocial characteristics? 2. What is the prognostic value of these clinical phenotypes for outcomes in pain and disability after THA?	- Sociodemographic and biomedical information (T0) - Pain-related cognitions and emotions (T0) - Traumatic experiences (T0) - Mental disorders (T0) - Self-efficacy (T0) - Social support (T0) - Perceived stress (T0) - QST (T0) - Other pain-related variables (T0)	Primary: HOOS Secondary: NPRS, PBM, SF-36, PSFS, GPE, Satisfaction, Muscle Strength	**Design**: Cross-sectional analysis of baseline data **Main statistical method**: Decision Tree learning
3	To identify pre- and early postoperative prognostic factors for long-term outcomes in pain and disability after THA in individuals with hip OA	1. To which extent do pre-operative traumatic experiences, mental disorders, pain-related cognitions and emotions, self-efficacy, social support, and central pain mechanisms predict long-term postoperative pain and disability after THA for hip OA? 2. To which extent do early postoperative pain-related cognitions and emotions predict long-term pain and disability after THA for hip OA?	- Pain-related cognitions and emotions (T0) - Traumatic experiences (T0) - Mental disorders (T0) - Self-efficacy (T0) - Social support (T0) - QST (T0)	Primary: HOOS Secondary: NPRS, PBM, SF-36, PSFS, GPE, Satisfaction, Muscle Strength	**Design**: longitudinal prospective **Main statistical method**: Gradient Boosting Algorithms
4	To identify postoperative clinical phenotypes based on biopsychosocial characteristics at different timepoints up to one year after THA for hip OA	1. Which postoperative clinical phenotypes exist based on biopsychosocial characteristics at different timepoints up to one year after THA for hip OA? 2. What is the relationship between preoperative and postoperative clinical phenotypes in individuals with hip OA and after THA? 3. To which extent do pre- and early postoperative factors predict the belonging to a clinical phenotype after THA?	- Sociodemographic and biomedical information (T2, T3, T4) - Pain-related cognitions and emotions (T2, T3, T4) - Traumatic experiences (T2, T3, T4) - Mental disorders (T2, T3, T4) - Self-efficacy (T2, T3, T4) - Social support (T2, T3, T4) - Perceived stress (T2, T3, T4) - QST (T2, T3, T4) - Other pain-related variables (T2, T3, T4)	Primary: HOOS Secondary: NPRS, PBM, SF-36, PSFS, GPE, Satisfaction, Muscle Strength	**Design**: longitudinal prospective **Main statistical method**: Recurrent Neural Networks

*Legend: OA = osteoarthritis, THA = total hip arthroplasty, QST = Quantitative Sensory Testing, NPRS = Numeric Pain Rating Scale, HOOS = Hip Disability and Osteoarthritis Outcome Score, PBM = Performance-Based Measures, SF-36 = 36-item Short-Form Health Survey, PSFS = Patient Specific Functioning Scale, GPE = Global Perceived Effect. Timepoints: one week before THA (T0), during the first week after THA (T1), and six weeks (T2), three months (T3), 12 months after THA (T4)*

### Participants

Two-hundred participants will be recruited from a secondary care setting at Hospital East-Limburg in Genk (Belgium) and the European Hip Clinic in Herselt (Belgium) starting from May 2021. Persons with a confirmed clinical or radiographic primary diagnosis of hip OA who are on the waiting list for a THA will be invited to participate in this study. Exclusion criteria are (1) rheumatic arthritis or other rheumatic diseases, (2) avascular necrosis or other pathological conditions explaining the symptoms, (3) neurological disorders (e.g., Parkinson’s disease, dementia…) significantly influencing the symptoms of hip OA, (4) revision THA, (5) a history of pathological fractures (e.g., osteoporosis, tumour…), (6) other planned surgical procedures during the follow-up period of twelve months (e.g., contralateral THA, TKA…). All participants will have to provide written informed consent before being included in the study.

### Procedure

Participants will be asked to complete a set of questionnaires in Qualtrics, an online GDPR-compliant platform (see Table 2 for the list of questionnaires used at the different timepoints). A semi-structured psychiatric interview will be conducted by a researcher that was trained by a psychiatrist to evaluate mental co-morbidities. A clinical psychologist (KB) is directly involved in the study and can be consulted by the researcher during the interview to obtain a second opinion if necessary. If any underlying psychological symptoms, traumatic experiences, or psychiatric symptoms are revealed by the Hospital Anxiety and Depression Scale (HADS), the Childhood Trauma Questionnaire (CTQ), the Traumatic Experiences Checklist (TEC) or the Simplified Mini International Neuropsychiatric Interview (MINI-S), this will be discussed with the participant, and their general practitioner will be notified by mutual agreement. Furthermore, the clinical psychologist can be contacted by mutual agreement with the participant for further clinical intake and referral to appropriate care if necessary. Furthermore, participants will undergo an extensive physical examination, including muscle strength testing, quantitative sensory testing (QST), and performance-based measures (PBM). Complete descriptions of the tests are presented here below.

**Table 2:** Overview of the assessments and time points

**Phenotyping variables or candidate prognostic factors**	**Variables**	**Timepoints**
Sociodemographic and biomedical information	Age, sex, height, smoking status, educational level	T0
	Body weight, marital status, employment status, number of comorbidities, number of physiotherapy sessions, sport	T0, T2, T3, T4
Pain-related cognitions and emotions	Fear-Avoidance Components Scale – Dutch (FACS-D) [51]	T0, T1, T2, T3, T4
	Tampa Scale for Kinesiophobia (TSK-17) [52]	T0, T1, T2, T3, T4
	Injustice Experience Questionnaire (IEQ) [53]	T0, T1, T2, T3, T4
Self-efficacy	General Self-Efficacy Scale (GSES) [54]	T0, T2, T3, T4
Social support	Groningen Orthopaedic Social Support Scale (GO-SSS) [55]	T0, T2, T3, T4
Perceived stress	Question from the Perceived Stress Scale (PSS) [56]	T0, T1, T2, T3, T4
Traumatic experiences	Childhood Trauma Questionnaire (CTQ) [57]	T0, T4
	Traumatic Experiences Checklist (TEC) [58]	T0, T4
Mental disorders	MINI-S DSM-V [59]	T0, T4
	MINI-S DSM-IV Suicidal risk [60]	T0, T4
	Hospital Anxiety and Depression Scale (HADS) [61]	T0, T1, T2, T3, T4
Quantitative Sensory Testing (QST)	Cold detection threshold (CDT) (local, remote)	T0, T2, T3, T4
	Warmth detection threshold (WDT) (local, remote)	T0, T2, T3, T4
	Cold Pain Threshold (CPT) (local, remote)	T0, T2, T3, T4
	Heat Pain Threshold (HPT) (local, remote)	T0, T2, T3, T4
	VAS 60 Temperature	T0, T2, T3, T4
	Temporal Summation of Pain (TSP)	T0, T2, T3, T4
	Conditioned Pain Modulation (CPM)	T0, T2, T3, T4
**Outcome variables**	**Variables**	**Timepoints**
Self-reported pain and disability	Hip disability and Osteoarthritis Outcome Score (HOOS) [62]	T0, T2, T3, T4
	Patient Specific Functioning Scale (PSFS) [63]	T0, T2, T3, T4
	Numerical Pain Rating Scale (NPRS) [64]	T0, T1, T2, T3, T4
Health-related quality of life	36-item Short-Form Health Survey (SF-36) [65]	T0, T2, T3, T4
Effect of THA on hip complaints in general	Global Perceived Effect (GPE) [66]	T0, T1, T2, T3, T4
Satisfaction with the outcome after THA	Satisfaction (NRS)	T0, T1, T2, T3, T4
Performance-based measures (PBM) [67]	30 s chair stand test (30s-CST)	T0, T2, T3, T4
	40 m fast paced walk test (40 m FPWT)	T0, T2, T3, T4
	10-step stair climb test (10-step SCT)	T0, T2, T3, T4
Muscle strength testing [68]	Isometric muscle strength of the hip flexors, extensors, abductors, adductors, external rotators	T0, T2, T3, T4

### Phenotyping variables/candidate prognostic factors

#### Sociodemographic and biomedical information

Participants will be asked to indicate their age, gender, height (m), body weight (kg), smoking status, educational level, marital status, and employment status. Self-reported height and body weight will be used to calculate Body Mass Index (BMI)(kg/m^2^). The number of comorbidities will be evaluated using a standardized self-created list of common comorbidities (heart disease, diabetes, psychiatric disorders, epilepsy, cancer, hypertension, major surgery in the past, osteoporosis, joint replacement, or other comorbidities). Additionally, participants will have to indicate whether they perform sports (activities that are intense enough so that one sweats at least to a slight degree from them) on a regular basis (yes/no), and whether they have previously received physiotherapy treatment for their hip complaints (yes/no). The number of physiotherapy sessions will be registered, as well as the type of physiotherapy exercises and treatments (e.g. education, strengthening exercises, cardiovascular exercise…). Finally, participants will be asked whether they received any psychological therapy during the follow-up period, and which type of psychological therapy they received.

#### Pain-related cognitions and emotions

Fear-avoidance will be assessed by the Dutch version of the Fear-Avoidance Components Scale (FACS-D) [51], which is a 20-item questionnaire. Each item is scored on a six-point Likert scale, resulting in scores ranging from zero (“completely disagree”) to five (“completely agree”). There is a maximum total score of 100, with higher scores indicating more fear-avoidance. Five severity levels have been proposed: subclinical (0–20), mild (21–40), moderate (41–60), severe (61–80), and extreme (81–100) [52]. The FACS-D has good reliability and validity in persons with chronic musculoskeletal pain, including persons with chronic hip pain [51].

Pain-related fear of movement and (re)injury will be assessed with the 17-item version of the Tampa Scale for Kinesiophobia (TSK-17) [53]. Each item is scored on a four-point Likert Scale, ranging from one (“strongly disagree”) to four (“strongly agree”). The total score ranges between 17 and 68, with higher values reflecting greater fear of movement. Measurement properties of the TSK-17 are sufficient in patients with chronic musculoskeletal pain [54–58].

Perceived injustice will be measured with the Injustice Experience Questionnaire (IEQ) [59]. The IEQ consists of 12 items, each item is scored from zero (“not at all”) to four (“all the time”). The total score ranges between zero and 48 and higher total scores reflect higher levels of perceived injustice. The validity of the IEQ is sufficient in patients with musculoskeletal pain [59].

#### Traumatic experiences

The Dutch version of the Childhood Trauma Questionnaire (CTQ) will be used to assess whether participants had a history of abuse or neglect in childhood [60, 61]. The CTQ consists of 25 items which evaluate childhood maltreatment across five dimensions: (1) physical abuse; (2) physical neglect; (3) emotional abuse; (4) emotional neglect; and (5) sexual abuse. Each item is scored on a five-point Likert scale, ranging from one (“never true”), to five (“very often true”). The total score ranges from 25 to 125, and each subscale score ranges from five to 25. The Dutch version of the CTQ was found to be valid and reliable [61].

The Traumatic Experiences Checklist (TEC) is a self-reported questionnaire to evaluate a persons’ history of traumatic experiences. The Dutch TEC will be used to evaluate a wide range of potential traumatizing experiences (29 types) [62]. The checklist covers traumatic experiences in six areas: (1) emotional abuse; (2) emotional neglect; (3) sexual harassment; (4) sexual abuse; (5) physical abuse; and (6) threat to life or bizarre punishment/intense pain. Furthermore, 11 items relating to family events are included, such as a divorce or loss of a significant other. Participants have to indicate whether or not they experienced a traumatic event, the age at onset, the duration of the trauma, and the subjective impact of the traumatic event, ranging from one (“no impact”) to five (“very severe impact”). The total score ranges from zero to 29 and indicates the number of traumatic experiences. Psychometric properties of the TEC were found to be good [63].

#### Mental disorders

The Mini International Neuropsychiatric Interview Simplified (MINI-S) for the Diagnostic and Statistical Manual of Mental Disorders 5 (DSM-5) will be used to identify psychiatric comorbidity [64]. The MINI-S is a brief semi-structured diagnostic interview for the major psychiatric disorders in the DSM-5. The MINI-S assesses the 17 most common disorders in mental health. Additionally, the suicidal risk module of the MINI-S DSM-4 will be administered [65, 66].

The Hospital Anxiety and Depression Scale (HADS) will be used to screen for symptoms of anxiety and depression. The HADS is a 14-item questionnaire evaluating symptoms of anxiety and depression without involving physical complaints [67]. One subscale covers symptoms of anxiety (HADS-A), the other symptoms of depression (HADS-D). Each item is scored from zero (”not applicable”) to three (”certainly applicable”). The maximum score on each subscale is 21, with higher values indicating more anxiety/depression symptoms. Both the anxiety and depression subscales have good psychometric properties in musculoskeletal pain populations [68].

#### Self-efficacy

The General Self-Efficacy Scale (GSES) is a self-reported questionnaire used to measure self-efficacy [69]. The GSES measures how a person generally copes with stressors/difficult situations in life. It consists of ten statements (optimistic “self-beliefs”) that ask about how people think and act in general. Items are scored on a four-point Likert scale.

#### Perceived stress

Perceived stress will be evaluated with a single question from the Perceived Stress Scale (PSS): “In the last month, how often have you felt nervous and stressed?” [70]. The question is scored on a five-point Likert scale, ranging from zero ‘never’ to four ‘very often’. The PSS has been found to be reliable and valid in different populations [71].

#### Social support

The Groningen Orthopaedic Social Support Scale (GO-SSS) is a self-reported questionnaire that has been developed to measure social support in patients with a THA or TKA [72]. Social support is measured through 12 statements, and each item is scored on a four-point Likert scale ranging from one (“never or rarely”) to four (“often”), resulting in a total score ranging from zero to 48. Validity and reliability of the GO-SSS were found to be sufficient for individuals undergoing total hip or knee arthroplasty [72].

#### Other pain-related variables

Participants will be asked to indicate their pain duration (months), use of pain medication (none, seldom, most days and/or nights, all days and/or nights), number of painful body regions (last week and last year) assessed with a self-created list of body regions, and the number of painful days last week.

#### Quantitative sensory testing (QST)

Quantitative sensory testing (QST) can be used to measure peripheral and central somatosensory function in persons with musculoskeletal pain [73, 74]. A Peltier-based computerized thermal stimulator (TSA II; Medoc Ltd, Ramat-Ishay, Israel) will be used to perform QST measurements. The TSA II uses thermal stimulation to assess the functionality of small nerve fibers and gain insight into the physiological and psychological processes underlying pain and sensation responses. The standardised QST protocol that is described below, including measures of local and widespread hypo- and hyperalgesia, temporal summation of pain, and conditioned pain modulation, will be performed in all participants. Heat and cold stimuli will be applied by means of a 30 × 30 mm thermode. The baseline temperature will be set to 32 °C for all these measurements.

The protocol of the German Research Network on Neuropathic Pain (DFNS) will be followed to determine thermal detection and pain thresholds [75]. Cold detection thresholds and warmth detection thresholds (in °C) will be assessed locally (at the most painful site of the hip) and remotely (at the volar aspect of the contralateral wrist) using a limits protocol (rate of 1 °C/s, interstimulus interval 4s). Cold pain thresholds and heat pain thresholds (in °C) will be assessed locally (at the most painful site of the hip) and remotely (at the contralateral wrist) using a limits protocol (rate of 1 °C/s, interstimulus interval 10s). Four trials will be performed for each threshold. The first trial will be used to give the patient a sense of what is expected. The average temperature of the last three trials will be calculated to define the specific threshold.

To determine the temperature that corresponds with a visual analogue scale (VAS) score of 60, a VAS Search protocol will be performed at the volar aspect of the contralateral wrist. The thermode will repeatedly heat up from the baseline temperature (32 °C) to a temperature between 39 and 50 °C. Participants will have to indicate their pain intensity for these stimulus intensities on a scale from zero, “no pain”, to 100, “worst imaginable pain”. Depending on their pain intensity, the thermode will heat up to a higher or lower temperature until the participant indicates a VAS of 60 out of 100. The temperature corresponding to a VAS 60 is determined to be used in the dynamic QST-protocols for the evaluation of temporal summation and conditioned pain modulation, with a maximum of 45 °C.

Temporal summation of pain will be assessed at the contralateral wrist using a two-minute tonic heat stimulus and participant-controlled temperature [76]. Participants are presented with a tonic heat stimulus and are instructed to maintain their initial sensation for two minutes by increasing or decreasing the temperature (rate of 1 °C/s) via the response unit. To quantify temporal adaptation and temporal summation of pain, the magnitude of temperature changes will be extracted and the areas under the curve will be calculated.

Conditioned pain modulation will be evaluated using a Dual-Thermode program with two heat stimuli [77]. The test stimulus will be a heat stimulus administered twice at the volar aspect of the contralateral wrist. Once on its own before administering the conditioning stimulus (interstimulus interval 10s), and once at the end of the conditioning heat stimulus at the volar aspect of the ipsilateral wrist. The conditioning stimulus will be a heat stimulus administered at the volar aspect of the ipsilateral wrist after first applying the test stimulus. The difference in pain intensity at the contralateral wrist during the stand-alone test stimulus and the test stimulus during the conditioning stimulus will be calculated. Pain intensity is assessed using a VAS ranging from zero to 100, where zero stands for “no pain”, and 100 for “worst imaginable pain”.

### Outcome variables

#### Disability

Perceived disability will be assessed with the Hip disability and Osteoarthritis Outcome Score (HOOS), a self-reported questionnaire for evaluating symptoms and disability in persons with hip complaints [78, 79]. The HOOS has been validated in Dutch [80] and consists of 40 items, divided into five subscales: symptoms (five items), pain (ten items), functioning in activities of daily living (17 items), functioning in sport and recreation (four items), and quality of life (four items). Each question is scored on a five-point Likert scale, in which a higher score reflects fewer complaints. The total score of the HOOS ranges from zero to 100, and a higher score indicates lower disability. A normalized score from zero to 100 is calculated for each subscale as well. The Dutch version of the HOOS was found to be valid and reliable in individuals with OA [81].

The Patient Specific Functioning Scale (PSFS) will be used to evaluate the functional status of the participants within the ICF activity and participation domain [82]. The participants will be asked to name the three most important activities that are difficult or impossible to perform due to the hip problems. The participant will have to score the degree of impairment in the activities on an 11-point NRS scale, ranging from zero “cannot perform the activity” to ten “can perform the activity just as well as before occurrence of the hip problem”.

The Osteoarthritis Research Society International recommended minimum core set of performance-based outcome measures (PBMs), including the 30 s chair stand test, the 40 m fast paced walk test, and the 10-step stair climb test, will be performed to evaluate physical function [83]. Movement quality during the performance-based tests will be assessed based on acceleration data measured with an Inertial Measurement Unit [84]. The 30-second Chair Stand Test will be used to assess sit-to-stand activity. The maximum number of chair stand repetitions within 30 s will be used as the outcome value. The 10-step Stair Climb Test will be used to assess ascending and descending stair activity. The time in seconds to ascend and descend ten stairs will be used as the outcome value. The 40-meter (4 × 10-meter) Fast Paced Walk Test will be used to assess short distance walking activity. The time in seconds to walk four times ten meters for a total of 40 m will be used as the outcome value.

#### Pain

The average pain intensity over last week and the current pain intensity will be assessed by the Numeric Pain Rating Scale (NPRS), an 11-point scale ranging from zero (“no pain”) to ten (“worst possible pain”)[85]. The NPRS has appropriate measurement properties in patients with musculoskeletal pain [86, 87].

#### Quality of life

The 36-item Short Form Health Survey will be used to evaluate health-related quality of life. The questionnaire is divided in eight health-related dimensions: physical functioning, role limitations due to physical problems, role limitations due to emotional problems, pain, mental health, energy, general health perception, and social functioning. A higher score indicates better health-related quality of life. The Dutch version of the SF-36 was found to be a valid and reliable instrument in individuals with different chronic diseases [88].

#### Muscle strength

Hip muscle strength will be measured with the MicroFet 2®, a handheld dynamometer. In the test protocol, the following muscle groups will be tested consecutively: abductors (side lying), adductors (supine), extensors (prone), hip flexors (sitting), external rotators (sitting). Each muscle group will be tested three times, with the best value of the three tests as the final result [89].

#### Other outcome variables

Satisfaction will be assessed with an 11-point Numeric Rating Scale (NRS), ranging from zero (“not satisfied”) to ten (“very satisfied”). The participant’s opinion about recovery after THA will be evaluated using the Global Perceived Effect score on a seven-point Likert scale, ranging from zero “a lot worse” to seven “a lot better” [90].

### Statistical analysis

Statistics will be performed in R (Version 3.6.3) using RStudio (2022.07.2). According to the different research questions and the type of data available, different statistical methods will be used (see Table 1). A sample size calculation was performed in R based on the third objective to identify pre- and early postoperative prognostic factors for long-term outcomes in pain and disability after THA for hip OA. A sample size of 200 participants will be needed for a prognostic model with 12 independent variables, a power of 80%, an effect size of 0.15, and a drop out of 20%.

For study 1, a LASSO regression (Least Absolute Shrinkage and Selection Operation) analysis will be used to assess the potential relationship between traumatic experiences and mental disorders on pain processing variables (QST) [91]. LASSO regression is a technique that extends the traditional linear regression by incorporating a regularization term that penalizes the size of the regression coefficients. This penalty aids in reducing model complexity and identifying the most essential predictors. When working with high-dimensional data containing numerous predictor variables, LASSO regression is particularly useful. LASSO is superior to traditional linear regression in its ability to prevent overfitting, enhance model interpretability, and reduce estimate variance.

If no suitable model (autocorrelation, residual distribution) can be found, a neural network will be used to answer this research question [92].

In study 2, since all pre-operative phenotyping variables will be included in the model, decision tree learning will be used to select the most relevant variables to include in the model to identify preoperative clinical phenotypes [93]. Decision tree learning is a type of machine learning algorithm that constructs a tree-like model of decisions. An important distinction between decision tree learning and traditional linear regression is that decision trees can deal with both categorical and continuous data, whereas linear regression requires that independent variables be continuous. In addition, decision trees can account for non-linear correlations between variables and interactions among predictors. Moreover, decision trees are easier to interpret than linear regression models. The structure of the decision tree is readily comprehensible, as are the rules for predicting outcomes.

If this approach is not successful, random decision forest will be used [94]. However, it is preferred to keep the model as simple as possible to ease the clinical interpretation.

For the study 3 gradient boosting algorithms will be applied to the dataset to select the best prognostic factors for long-term outcomes in terms of pain and disability [95]. Gradient boosting techniques are a type of ensemble learning that combines numerous weak predictors to create a more accurate one. It adds decision trees to the model in an iterative manner, with each new tree aiming to correct the errors of the preceding trees. Gradient boosting algorithms are especially effective when working with complex data and are frequently capable of achieving high accuracy on a wide variety of problems compared to stepwise linear regression. Previous contralateral THA will be included as a covariate in the analysis to account for possible bias due to knowledge of surgical procedures and postoperative recovery course.

Finally, for study 4, recurrent neural networks (RNN) will be applied to identify postoperative clinical phenotypes based on the multiple data points (repeated measurements during T2, T3, and T4) [96]. RNNs are a sort of neural network architecture that can process sequences of inputs, such as time series. They can keep an internal memory of previous inputs and use it to make predictions about future inputs. RNNs are particularly good for modelling complex temporal interactions. When working with sequential data, RNNs tend to be more flexible and robust than stepwise regression. They are able of modelling complex temporal relationships and less susceptible to overfitting.

## Discussion

The present study will contribute to the knowledge needed to improve pain and disability in individuals with hip OA and after THA. From a clinical point of view, the identification of clinical phenotypes before and after THA, based on a set of biopsychosocial characteristics, may represent a fundamental step towards the development of pre- and postoperative precision medicine pathways for individuals with hip OA undergoing THA. From a scientific perspective it can provide a basis for subgroup analyses in clinical trials, or it can facilitate the implementation of enrichment strategies in randomized controlled trials. Furthermore, prognostic factors for outcomes in pain and disability after THA will contribute to the improvement of outcomes in pain and disability by enabling healthcare providers to identify persons at risk of poor outcome, and consequently increasing options for shared decision making, expectation management and precision medicine [18].

This longitudinal prospective cohort study has several strengths and innovative aspects. First and foremost, phenotyping variables across all domains of the biopsychosocial framework are included, which has been a missing element in studies investigating clinical phenotypes in individuals with hip OA [15, 97]. Moreover, except for the sociodemographic and biomedical variables, all phenotyping variables are modifiable and treatable and thus of great clinical importance. An important innovative aspect is that this will be the first study in individuals with hip OA to include both self-reported biopsychosocial characteristics and mechanistic pain profiling using QST. This allows us to investigate whether clinical phenotypes are characterized by different pain mechanisms, and consequently may need different treatment approaches. Additionally, the primary outcome measures are situated within the clinically relevant activity and participation domain of the International Classification of Functioning, Disability and Health, and include both self-reported and performance-based outcome measures. Moreover, the inclusion of secondary outcome measures such as quality of life, outcome satisfaction, global perceived effect, and muscle strength, in addition to outcome measures related to pain and disability, also contributes to the clinical significance of this study. This allows assessment of both subjective parameters of patient satisfaction and objective improvements in function. Another strength of this study is the longitudinal prospective design, which makes it possible to evaluate the evolution of pre- and postoperative clinical phenotypes [98]. The longitudinal design also allows an extensive follow-up assessment, including early postoperative, short-term postoperative, and long-term postoperative measurements. To our knowledge, no other studies have included early postoperative measurements of pain-related cognitions and emotions in the week following surgery. Most studies focus on preoperative prognostic value of candidate prognostic factors; however, postoperative values might be interesting in prognosis as well. For example, short-term postoperative self-efficacy seems to be a better prognostic factor than preoperative self-efficacy in patients with THA [99]. Finally, we aim to unravel the prognostic role of biopsychosocial factors that are not yet investigated in THA research, including perceived injustice and traumatic experiences. The prognostic factors that will be identified are potential predictors of treatment effect and can improve the accuracy of prognostic models for outcome prediction after THA [100]. In follow-up of the current trial, future randomized controlled trials should further elaborate the causal relationships between identified prognostic factors and treatment outcomes in order to identify predictors of treatment effect. In a future RCT, phenotyping at baseline could be performed in an enrichment design to selectively enroll participants with a specific clinical phenotype. Participants with this clinical phenotype could then be randomized into two groups, receiving either specific treatment targeting the phenotypic prognostic factors or standard care as a control intervention. Consequently, the effect of the intervention on the phenotypic prognostic factor and on the outcome after THA should be compared between the intervention and the control group to determine whether the phenotypic prognostic factor is also a predictor of treatment effect. This might provide information to develop individually-targeted treatment pathways for persons with hip OA, which can further improve phenotype-specific treatments. The combination of precision medicine pathways and careful selection of appropriate candidates for THA might optimize outcomes in pain and disability both before and after THA [1].

In addition to the strengths and innovative aspects of this study, there are also some practical issues and potential limitations to consider. In a recent systematic review [101], we investigated contextual prognostic factors for outcomes in the International Classification of Functioning, Disability and Health activity and participation domain after THA in individuals with hip OA. In this review, the main issues in prognostic factor research in individuals after THA for hip OA were discussed. Currently, most studies on prognostic factors for outcomes after THA have a high risk of bias, with mainly problems regarding study attrition and confounding. Therefore, an important practical consideration for this study will be to limit drop-out and accommodate study attrition of participants. The timepoints for follow-up measurements will therefore be scheduled together with the postoperative monitoring appointments with the surgeon to reduce the burden on participants. Furthermore, confounders will be clearly defined and measured using valid and reliable tools, and will be appropriately accounted for in the statistical analysis. One of the limitations of this study will be that generalizability will be limited to individuals with hip OA eligible for THA, and individuals undergoing direct anterior approach THA, since there is a significant preselection of individuals with hip OA undergoing direct anterior approach THA. Furthermore, no external validation is included in this study protocol. When a certain subgroup classification or prognostic factors will be identified, replication of the findings will be necessary in another sample.

## Data Availability

The datasets used and/or analysed during the current study will be available from the corresponding author on reasonable request and with permission of Hasselt University.

## Abbreviations

OA: Osteoarthritis
THA: Total Hip Arthroplasty
LASSO: Least Absolute Shrinkage and Selection Operator
QST: Quantitative Sensory Testing
TKA: Total Knee Arthroplasty
PBM: Performance-Based Measures
BMI: Body Mass Index
FACS-D: Dutch version of the Fear-Avoidance Components Scale
TSK-17: Tampa Scale for Kinesiophobia 17-item version
IEQ: Injustice Experience Questionnaire
CTQ: Childhood Trauma Questionnaire
TEC: Traumatic Experiences Checklist
MINI-S: Mini International Neuropsychiatric Interview Simplified
DSM: Diagnostic and Statistical Manual of Mental Disorders
HADS: Hospital Anxiety and Depression Scale
HADS-A: Hospital Anxiety and Depression Scale subscale Anxiety
HADS-D: Hospital Anxiety and Depression Scale subscale Depression
GSES: General Self-Efficacy Scale
PSS: Perceived Stress Scale
GO-SSS: Groningen Orthopaedic Social Support Scale
VAS: Visual Analogue Scale
HOOS: Hip disability and Osteoarthritis Outcome Score (HOOS)
PSFS: Patient Specific Functioning Scale
NRS: Numeric Rating Scale
SF-36: 36-item Short Form Health Survey
